# Antibiotic Application and Resistance in Swine Production in China: Current Situation and Future Perspectives

**DOI:** 10.3389/fvets.2019.00136

**Published:** 2019-05-17

**Authors:** Hong Yang, Lisa Paruch, Xunji Chen, André van Eerde, Hanne Skomedal, Yanliang Wang, Di Liu, Jihong Liu Clarke

**Affiliations:** ^1^Norwegian Institute of Bioeconomy Research, Ås, Norway; ^2^Department of Geography and Environmental Science, University of Reading, Reading, United Kingdom; ^3^Xinjiang Academy of Agricultural Sciences, Urumqi, China; ^4^Heilongjiang Academy of Agricultural Sciences, Harbin, China

**Keywords:** antibiotics, antimicrobial resistance, bacteria, China, human and animal health, swine production

## Abstract

To meet increasing demand for animal protein, swine have been raised in large Chinese farms widely, using antibiotics as growth promoter. However, improper use of antibiotics has caused serious environmental and health risks, in particular Antimicrobial resistance (AMR). This paper reviews the consumption of antibiotics in swine production as well as AMR and the development of novel antibiotics or alternatives in China. The estimated application of antibiotics in animal production in China accounted for about 84240 tons in 2013. Overuse and abuse of antibiotics pose a great health risk to people through food-borne antibiotic residues and selection for antibiotic resistance. China unveiled a national plan to tackle antibiotic resistance in August 2016, but more support is needed for the development of new antibiotics or alternatives like plant extracts. Antibiotic resistance has been a major global challenge, so international collaboration between China and Europe is needed.

## Introduction

**Figure 1:**
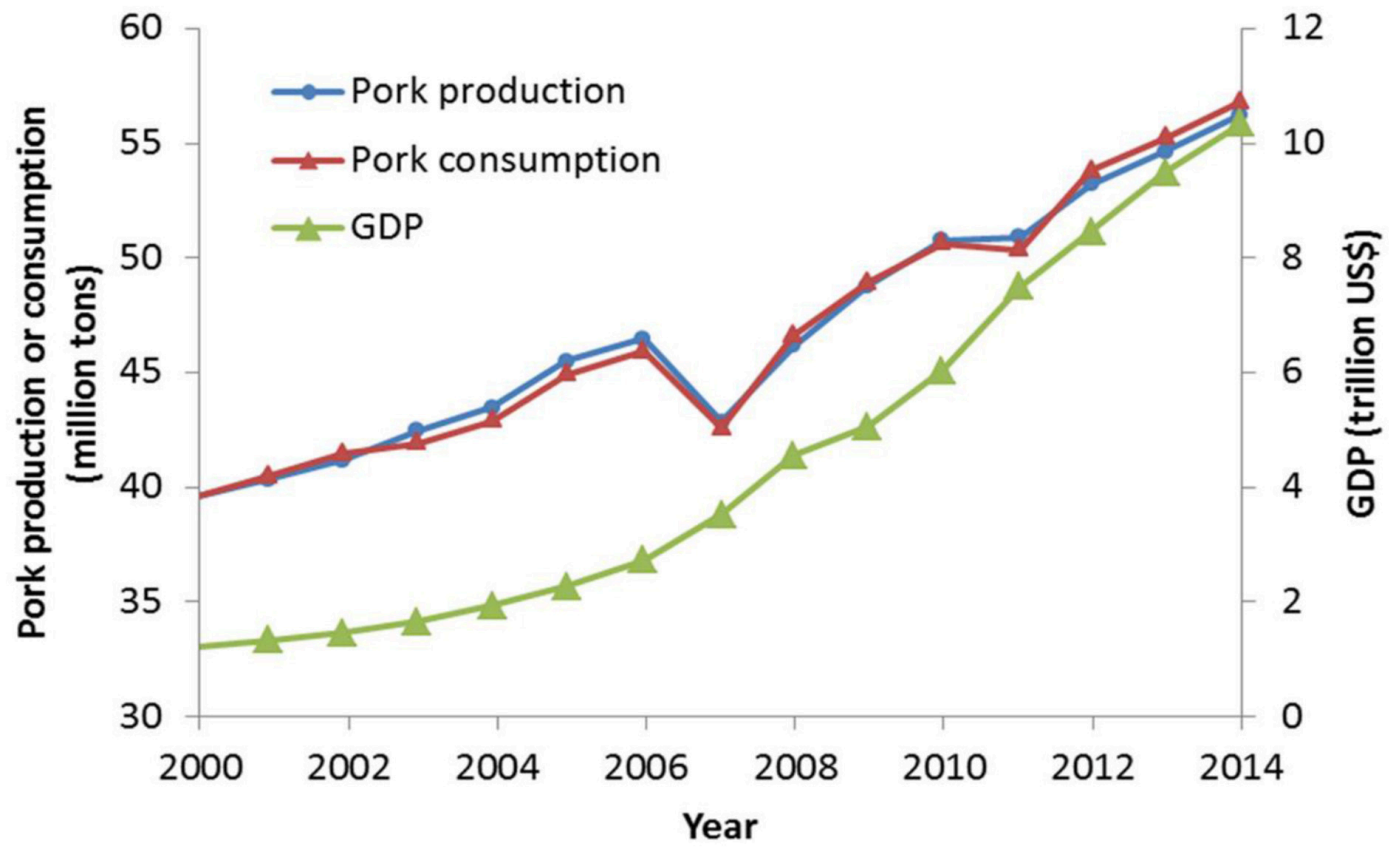
Gross domestic production (GDP), pork production and consumption in China from 2000 to 2014 (data source: World Bank http://data.worldbank.org/country/china and USDA Foreign Agricultural Service http://www.fas.usda.gov/).

Over the last decades, China's economy has grown very quickly. The gross domestic product (GDP) increased from 1.21 trillion US$ in 2000 to 10.35 trillion US$ in 2014 (Figure 1, World Bank, 2016). During the same period, Chinese production of meat, eggs, and milk has rapidly increased, and this will continue—especially for pork (1–3). Pork is one of the most important sources of animal protein in the country, and its production has jumped from around 40 million tons in 2000 to approximately 56 million tons in 2014 (Figure 1, USDA, 2016). The effects of the global financial crisis in 2007 and swine flu in 2011 caused an AMRupt production decline in these 2 years. However, production of swine quickly rebounded in the subsequent years. Concurrently, China's pork consumption increased from 2000 to 2014, with some drops in 2007 and 2011. Since 2012, pork consumption has been slightly higher than production, indicating that the pork demand of Chinese consumers has exceeded the domestic production.

Along with a rapid increase in pork production, both the number and the size of intensive swine farms have grown. The number of big farms with thousands of swine has increased markedly. The percentage of big swine farms, with herd sizes of more than 3,000, increased from 5% in 2003 to 14% in 2010. In the same period, the proportion of small farms, with herd sizes of less than 50, nearly halved, from 71% to 36% (China Animal Industry Yearbook 2004–2011).

Several recent studies detail antibiotic use in animal production (3–6) and the risk this poses in the form of antibiotic resistance (7–9). The current study focuses on the important emerging public health challenges as a result of overuse or abuse of antibiotics in swine production in China. It also outlines the future challenges for the new antibiotics and alternatives.

## Antibiotic Usage in Swine Production in China

With the shift from small to large swine feeding operations and the increase in overall pork production, there is growing concern about the adverse consequences such as swine health and welfare, disease spreading of large-scale animal production (1). Because the high density of animals in big swine farms exacerbates the risk of quick spread of infectious diseases, farmers in China have responded by using higher amounts of antibiotics. This, in turn, has led to growing concerns regarding overuse and abuse of antibiotics for intensive swine production, especially the health risks (3, 10, 11).

Penicillin was discovered in the 1940s. Since then, antibiotics have changed the treatment of bacterial infections for both humans and animals. Antibiotics were first added to feed for broiler poultry to prevent microbial diseases in the 1940s (12). They were then rapidly used for the same purpose in feed for other food animals, first in the USA and later in other developed countries and developing countries such as China (2). Antibiotics can aid in different ways. When antibiotics are used at low (sub-therapeutic) levels in feed, they can improve growth rate by reducing mortality and disease. Thus, conversion of feed to weight gain becomes more efficient. Antibiotics can further prevent disease at intermediate levels, whereas high (therapeutic) levels of antibiotics are used to treat diseases (13–15). Antibiotics are widely used in animal husbandry as low-cost growth promoters in more than half of the world's countries (7).

As the world's largest pork producer and consumer, China uses a massive amount of antibiotics to support its production (3). Some studies have been conducted to identify the antibiotics used in China's pig farms (16–23). These studies report the extensive use of the major antibiotic classes of sulphonamides, tetracyclines, fluoroquinolones, macrolides, and β-lactams.

Antibiotics have been widely adopted for use in food animals, but reliable data about the quantity and patterns of use (e.g., dose and frequency) for food animals alone are not easily available in China or other developing countries. It is very challenging to make an accurate calculation of antibiotic use in food animals. Studies have adopted different classifications for therapeutic use, nontherapeutic use, or a combination of the two. Most available data lack clear definition of therapeutic vs. nontherapeutic uses, and this ambiguity clearly erodes reliability (24). Based on models developed from American data (25), Krishnasamy et al. (24) estimated that 38.5 million kg of antimicrobials were consumed in China's pork and poultry production in 2012. Among all antibiotics, tetracyclines are the most widely consumed in swine production. Zhang et al. (23) performed a market survey on the usage of the 36 main antibiotics in China including sulfonamides, tetracyclines, fluoroquinolones, macrolides, β-lactams (penicillins and cephalosporins), chloramphenicols, lincomycin, and others. They found that the total amount of antibiotics used for China's swine farming was 48.4 million kg in 2013, which is higher than the result of Krishnasamy et al. (24). Of all antibiotics consumed in China's swine farming, fluoroquinolones and β-lactams contributed more than half.

Moreover, there is a clear geographic heterogeneity for antibiotic consumption in China. Antibiotic consumption hotspots appear in Southwest China (Sichuan), Central China (Hunan), North China (Henan and Hebei) and the southeast coast (Fujian, Guangdong and Guangxi) in China. In particular, Sichuan province has the highest swine density and therefore carries the most serious risks to environment and health (23). Other areas have also seen significant developments in recent years. For example, Xinjiang Uyghur Autonomous Region, the provincial level region with the largest area in Northwest China, is located in the center of the Eurasian continent. It is in the core area of “The Silk Road Economic Belt” and plays an important role in this program. Pork production in Xinjiang increased from 0.025 million tons in 1978 to 0.231 million tons in 2010 (Statistical Yearbook of Xinjiang in 2011). Despite a lack of data on antibiotic consumption in swine farms in Xinjiang, the concentration and detection rate of antibiotic residue in swine manure samples were higher than those of chicken manure and cow dung. The concentration of tetracycline in swine manure was highest, followed by sulfonamides and quinolones (26, 27). Additionally, international trade with Central Asian and European countries is increasing along the Silk Road, which may worsen the spread of antibiotics. In Lake Aibi, 12 species of 14 kinds of antibiotics were detected and detection rates of four kinds of antibiotics were 100% in water samples, with highest average concentration of 54.37 ng L^−1^ (28, 29).

China is tackling the overuse of antibiotics and the AMR problem using different approaches, including educating farmers about AMR caused by excessive use of antibiotics in animal farming, swiftly banning the use of colistin as a feed additive in animal production (30), reducing the list of approved antibiotics for animal application, promoting the use of alternative feed additives such as organic acids (e.g., Selko®-pH, http://selko.com), improving the management of animal husbandry and animal welfare, and law enforcement accompanied by an effective surveillance system [(3), http://www.moa.gov.cn/].

## Antibiotic Resistance and the Risk to Human Health

**Figure 2:**
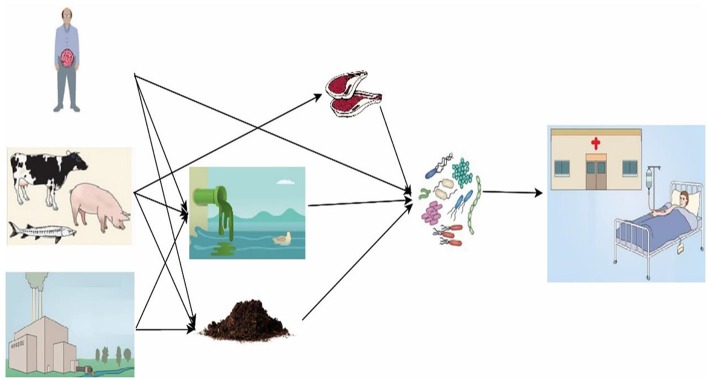
Expected fate, transport, and exposure pathways for antibiotics and the spread of antibiotic resistome. Antibiotics from human and veterinary drugs, growth promoter for animal husbandry and aquaculture, and improper release during pharmaceutical production are released into water and soil. Manure containing antibiotic resistome may be carelessly used for crop production. Antibiotic resistome can remain in meat and the bacteria can be further spread to humans. People take up antibiotics and resistome develops in their guts [modified from Song and guo (35) and Berendonk et al. (36)].

The overuse and abuse of antibiotics cause environmental pollution, for example the contamination of manure, soil and water (10, 31). Worse, improper use of antibiotics brings risk to human health through food-borne antibiotic residues and selection for AMR, and a greater ability of certain bacteria to resist the effect of antibiotic treatments (7). The causes of AMR are complex, but there is growing scientific evidence suggesting that low-dose, prolonged courses of antibiotic use for animal husbandry accelerated the emergence and spread of resistant bacteria (32–34). In food animal husbandry, AMR can spread not only by direct contact, but also indirectly (Figure 2). Direct effects are those that can be causally linked to contact with antibiotic-resistant bacteria from swine. Indirect effects are those that result from contact with resistant organisms that have been spread through food, water, and animal waste application to soil (37).

Many antibiotic classes are used in both swine husbandry and human health care. Therefore, the emergence and spread of resistance to these antibiotics will likely limit the therapeutic options for human diseases. Even worse, this kind of AMR can prolong illness and cause serious disability and ultimately death (32, 33).

In the last decades, AMR has become a global challenge for human health and welfare. In particular, it is a serious problem in China where antibiotics have been overused or misused in livestock husbandry and human health care (38–41). For example, the OqxAB efflux pump, encoded by the genes *oqxA* and *oqxB*, has been found to be one of the mechanisms of plasmid-mediated quinolone resistance (PMQR) (42–44). Zhao et al. (45) investigated the prevalence and dissemination of *oqxAB* in *Escherichia coli* (*E. coli*) isolates from swine, their environment and farmworkers in China. The *oqxA* gene was present in around 39.0% of *E. coli* isolates. About 46.3% of *E. coli* isolates from swine farms were positive for *oqxA*. Approximately 43.9% of *E. coli* isolates from the swine farm environment were also positive. In addition to animal *E. coli* isolates, *oqxAB* was found in 30.3% of human commensal *E. coli* isolates. Because these farmworkers were without previous antimicrobial treatment or hospital admission, this indicated the transmission of *oqxAB* to humans. Compared with results from Sweden (1.8%) and South Korea (0.4%) (46, 47), the prevalence of *oqxAB* in *E. coli* isolates was much higher (39.0%) in China (45).

A further example has been reported by Zhang et al. (48), who researched the occurrence of the *aac* (*3*)*-IV* gene, which confers resistance to apramycin, an antibiotic used in agriculture but not for humans, in Northeast China. Unfortunately, they found workers who carried apramycin resistance genes in all swine farms where apramycin was used as an antibiotic growth promoter. The same was present in swine isolates. Similarly, Ho et al. (49) investigated gentamicin resistance in Hong Kong. They found that 84.1% of human samples and 71.4% of swine samples contained the *aaaC2* gene for gentamicin resistance. Polymyxin resistance was identified as being due to the plasmid-mediated *mcr-1* gene (50). Liu et al. (51) investigated the *mcr-1* gene in swine, pork and inpatients in five provinces in China during the period 2011–2014. They found *mcr-1* in *E. coli* isolates collected from 17.7% of pork samples, 20.23% of swine samples, and 1.40% of inpatient samples with infection. Similar studies have also been conducted in Xinjiang. For example, Xia et al. (52) collected 543 fecal samples from a large-scale swine farm and isolated 454 *E. coli* isolates. They found that 64.5% of the *E. coli* isolates showed resistance to 3–9 antimicrobials, especially to ampicillin and amoxicillin.

## The Development of New Antibiotics

Concern about antibiotic resistance has escalated in the last years. In 1986, Sweden became the first country in the world to ban the use of some antibiotics in animal feeds (53). In 2006, European Union (EU) member nations started to ban all antibiotic growth promoters according to EC Regulation No. 1831/2003 (14). As the largest developing country with a growing demand for meat protein, China has not yet completely prohibited the use of antibiotics as growth promoters. Considering the big risk for antibiotic pollution in the environment (soil and water) and potential resistance, more research is urgently needed for the development of new antibiotics or, ideally, alternatives.

### New Antibiotics

During the past two decades, efforts to develop new antibiotics have met with some success (54). However, due to their much higher costs compared to the older antibiotics, many have been gradually pulled from the market. Therefore, new antibiotics are still needed to tackle the worsening risk of antibiotic resistance.

Several approaches have been applied to identify new antibiotics or augment currently licensed antibiotics: (1) natural or synthetic compounds as inhibitors of multidrug efflux pumps, (2) small-molecule inhibitors of bacterial transcription factors, and 3) antisense inhibition of multidrug transporter genes using licensed drugs (55–59). As alternatives to antibiotics, use of bacteriophage and plant extracts has also been investigated, which will be discussed in the next section.

By deleting or inactivating specific genes, researchers found some putative new targets, for example reducing the virulence of pathogens (60, 61). Quorum sensing (QS) or other bacterial signaling systems have also been identified as new targets for antibiotic molecules (62, 63). *In-silico* and *in vitro* high-throughput screening of small-molecule and compound libraries have also been increasingly used. Some agents have been in Phase 1 of clinical trials (64). In 2015, Ling et al. (65) discovered a “resistance-free” teixobactin in a screen of uncultured soil bacteria sample. Experiments confirmed no mutants of *Staphylococcus aureus* or *Mycobacterium tuberculosis* resistant to this teixobactin. Hopefully, this study will start an innovative approach to expanding the pool of natural antibiotics (66). Recently, a new class of antibiotics—arylomycins—was reported (67). The arylomycin G0775 showed activity against multi-drug resistant Gram-negative clinical bacterial pathogens by inhibiting the essential bacterial type I signal peptidase (which is a novel antibiotic target) through an unknown mechanism as described by Smith et al. (67). Further investigation will hopefully reveal the molecular mechanism underlying this novel class of antibiotics originating from natural products. Efforts will be made to identify and characterize more novel natural products to tackle AMR and problems caused by over-application of antibiotics in swine production.

### Plant Extracts—a Promising Alternative

In addition to searching for new antibiotics, alternatives/replacements have received growing attention in the last decades (14, 68). Researchers have explored various kinds of alternatives to animal antibiotics: feeding enzymes, immunity modulating agents, bacteriophages and their lysines, antimicrobial peptides, probiotics, prebiotics, synbiotics, inhibitors targeting pathogenicity, plant extracts and others (14, 69–75). In China, herbs and their extracts have been widely used in traditional medicine for centuries before the introduction of western medicine. Youyou Tu, from the China Academy of Traditional Chinese Medicine in Beijing, was awarded the 2015 Nobel Prize in Physiology or Medicine for her discovery of artemisinin (qinghaosu) extracted from *Artemisia annua* L. (76). Her work was inspired by the Chinese traditional medical book *Prescriptions for Emergencies* by Ge Hong (284–346 CE) (77). Compared to other antibiotic alternatives, therefore, plant extracts have received more attention and support in China.

Natural plant products and their derivatives have been explored for their antimicrobial, anti-inflammatory, anti-oxidative, and anti-parasite properties (78–84) (Table 1). A good example is garlic extract, which is widely considered as one of the most effective antibiotic agents (86). In addition, *Areca catechu* is a rich source of compounds with anti- quorum sensing (QS) properties (87). Some studies also found that *P. aeruginosa* genes controlled by QS could be inhibited by the isothiocyanate iberin from horseradish and ajoene from garlic (88, 89). When they are combined with tobramycin, ajoene and horseradish juice extracts function as a synergistic antibacterial (90). Extracts of the genus *Paeonia, one* of the most important sources of drugs in Chinese traditional medicine, can inhibit *C. albicans* growth (91). Extracts from *Fructus psoraleae, Folium eucalypti globuli* and *Achillea millefolium*, anti-dermatophitic compounds, have been used to treat different ailments such as dermatomycosis in Chinese traditional medicines (92, 93).

**Table 1:** Antibiotic alternatives: plant extracts.

**Plant**	**Effect observed**	**References**
Aged garlic extract, allicin	Improved growth performance, nutrient digestibility, intestinal microbial balance, immune response and meat quality in finishing pigs	(82)
*Camellia sinensis*	Improved gut health of post-weaning piglets and protection from *E.coli* challenge	(81)
Cinnamon essential oils, Cinnamaldehyde	Antimicrobial activity and improved immune response against e.g., *Salmonella typhimurium* in swine intestine	(83)
Carvacrol, cinnamaldehye, eugenol, etc.	Anti-inflammatory effects on porcine alveolar macrophages	(78)
*Capsicum* oleoresin, turmeric oleoresin, garlicon	Improved gut health and reduced frequency of diarrhea in weanling pigs	(79, 80)
*Agrimonia procera*	Growth performance, increased immune response and antioxidative effects in piglets	(84)
Chinese traditional herbal medicine (CTHM)	Beneficial effects on swine growth with improved final live weight, general digestibility and nitrogen retention	(85)

Diarrhea is a common cause of intestinal diseases in children and animals including swine (94). Some studies have been conducted to find plant extracts for inhibiting the proliferation of *E. coli*. Khan et al. (95) found that pathogenic strains of *E. coli* are sensitive to the extracts of three plants (*Acacia nilotica, Syzygium aromaticum* and *Cinnamum zeylanicum*). Herb extracts from *Pulsatilla chinensis, Sophora flavescens, Phellodendron amurense, Radix Astragali* and *Codonopsis pilosula* (Franch) Nannf have been used to treat diarrhea of piglets in Chongqing, Southwest China (96). Because of the influence of harvesting method and other unknown factors (97), plant extracts have been limited by their variability (98). The current high cost also limits the wide use of herb extracts, but the further development of herb extracts may reduce the cost and expand their application in developing countries.

Xinjiang is one of the Chinese regions with high biodiversity. The flora include common bitter beans, *Cynomorium, Ephedra, Ferula*, liquorice, snow lotus, sea buckthorn, and others. Among these, bitter beans have antibacterial ingredients (99). Horse grass contains alkaloids that, when drunk, can inhibit the function of malignant tumors. Xinjiang *Lithospermum* and liquorice contain glycyrrhizinate, flavonoids and other medicinal ingredients. These plant ingredients have antibacterial effects on *E. coli*, paratyphoid *Salmonella, Staphylococcus aureus, Bacillus subtilis* and other common pathogens (100).

### Future Perspective and Conclusions

Global organizations and developed countries have paid increasing attention to tackling the great risks of overuse and abuse of antibiotics and antibiotic resistance (101). For example, the World Health Assembly (WHA) commissioned the WHO to deliver a global action plan on antibiotic resistance in May 2014. The British government sponsored the £10 million Longitude Prize for the best solution for the resistance problem in June 2014. The President's Council of Advisors on Science and Technology in the USA released a report on antibiotic resistance in September 2014.

Slower than many European countries and the USA, China unveiled a national plan to tackle antibiotic resistance in August 2016 (102). The plan highlights the importance of reducing use of antibiotics in China's livestock husbandry. However, the implementation details of the plan are still unclear. Punishment for violations is still lacking. As for many action plans and laws in China, strict implementation is extremely important for reducing the use of antibiotics (103, 104). The plan also emphasizes the development of new antibiotics. As stated above, the high price of new antibiotics and alternatives limits their development (54). In the action plan, the funding source for discovery of new antibiotics or alternatives, for example from government or industry, is still unclear. Antibiotics have been widely overused and abused in Chinese swine farms to prevent diseases. However, it is more important to improve the sanitation and hygiene conditions of swine farms. Rather than using antibiotics, some measures should be applied to improve the health and well-being of swine, in particular reducing animal overcrowding, and controlling facility temperature and ventilation. In addition to the swine farmers, joint efforts from government, academia and veterinary professionals are indispensable.

Antibiotic resistance has become a world-wide challenge and therefore international collaboration is increasingly crucial. International collaboration between the world's largest antibiotics consumers, China and Europe, is indispensable to tackle the AMR problem. The One Health approach is of importance to achieve a sustainable and effective management of AMR by joint efforts of the international community with involvement of all stakeholders.

## Author Contributions

JC and DL designed the review, contributed to writing and editing. HY, LP, XC, AvE, HS, and YW contributed to writing, while JC is responsible for submission.

### Conflict of Interest Statement

The authors declare that the research was conducted in the absence of any commercial or financial relationships that could be construed as a potential conflict of interest.
